# Effectiveness of a Single Functional Neurology Intervention on Primitive Reflex Integration Dysfunction in Children

**DOI:** 10.1002/ccr3.70797

**Published:** 2025-08-14

**Authors:** Jorge Rey‐Mota, Guillermo Escribano‐Colmena, Athanasios A. Dalamitros, David Martín‐Caro Álvarez, Vicente Javier Clemente‐Suárez

**Affiliations:** ^1^ Faculty of Medicine, Health and Sports Universidad Europea de Madrid Madrid Spain; ^2^ Laboratory of Evaluation of Human Biological Performance, School of Physical Education & Sport Sciences Aristotle University of Thessaloniki Thessaloniki Greece; ^3^ Toledo Physiotherapy Research Group (GIFTO), Faculty of Physiotherapy and Nursing of Toledo Universidad de Castilla‐La Mancha Toledo Spain; ^4^ Grupo de Investigación en Cultura, Educación y Sociedad Universidad de la Costa Barranquilla Colombia

**Keywords:** functional neurology, grasp reflex, Moro reflex, primitive reflex, spinal galant reflex, tonic labyrinthine reflex anterior, tonic labyrinthine reflex posterior

## Abstract

A single session of functional neurology led to the integration of multiple retained primitive reflexes in a child, resulting in improved motor coordination and cognitive function. This case highlights functional neurology as a promising treatment for neurodevelopmental challenges.

## 1 Introduction

Primitive reflex integration dysfunction (PRID) refers to the condition where primitive reflexes, which are automatic movements seen in newborns, are not properly integrated as the child grows. These reflexes are crucial for survival in infancy and should typically diminish or transform into more complex motor patterns by the age of one. However, in some cases, these reflexes persist, leading to various developmental issues. PRID can contribute to a range of difficulties, including poor motor coordination, balance problems, and learning disabilities [1]. Primitive reflexes are involuntary motor responses originating in the brainstem that are present after birth and play a vital role in early child development [2]. These reflexes, such as the Moro reflex, palmar grasp reflex, and asymmetric tonic neck reflex, are essential for a newborn's survival and initial interaction with the environment [3]. However, the persistence of these reflexes beyond infancy can indicate central nervous system dysfunction and lead to developmental delays and disorders [4]. The incidence of PRID varies, but it is notably higher in children with neurodevelopmental disorders, such as autism spectrum disorder and attention deficit hyperactivity disorder (ADHD), affecting their daily functioning and academic performance [3]. Research indicates that children with persistent primitive reflexes may struggle with motor control, balance, and coordination, impacting their ability to engage in typical childhood activities and learning processes [1].

Functional neurology is gaining recognition as an effective approach for addressing various pathologies, including primitive reflex integration dysfunction. By integrating principles from traditional neurology with functional medicine, this field emphasizes the interconnectedness of neurological and systemic health. Functional neurology focuses on individualized treatment plans derived from comprehensive neurological assessments to restore balance and enhance overall health outcomes [5]. Recent advancements demonstrate its potential in treating conditions inadequately managed by conventional methods, providing new hope for patients with chronic and complex issues [6]. Previous research indicates that functional neurology interventions can improve quality of life by alleviating symptoms associated with neurological dysfunctions [7]. This approach is particularly promising in cases where traditional treatments have failed, offering a comprehensive and holistic method to patient care. Furthermore, studies have shown that such interventions can lead to significant improvements in both neurological function and overall patient well‐being, highlighting the importance of continued research and application in this emerging field [8].

The present study aimed to evaluate the effectiveness of a single functional neurology intervention in integrating primitive reflexes in a pediatric patient. Despite growing interest in functional neurology, there remains a paucity of empirical evidence supporting its application in treating PRID. This case study seeks to fill this gap by providing quantitative data on the improvement of specific primitive reflexes following treatment. The primary objective is to determine whether a targeted functional neurology intervention can significantly reduce the presence of primitive reflexes in a child with PRID. We hypothesize that the intervention will result in measurable improvements, as evidenced by lower scores on reflex integration assessments; thereby demonstrating the potential of functional neurology as a viable treatment option for PRID.

## 2 Case History/Examination

The patient was a 7‐year and 2‐month‐old female presenting with retained primitive reflexes, including the Asymmetrical Neck Reflex (ANR), Symmetrical Neck Reflex (SNR), Tonic Labyrinthine Reflex (TLR, anterior and posterior), Spinal Galant Reflex, Grasp Reflex, Moro Reflex, and Paralysis by Fear Reflex. The patient had difficulties in motor coordination, balance, and cognitive performance, indicating potential developmental delays linked to these retained reflexes. The reflexes were assessed based on their degree of integration, with scores ranging from 0 (integrated) to 4 (not integrated). Initial evaluations revealed that several reflexes, particularly the ANR (3/4), SNR (3/4), and TLR (2/4), were not integrated, contributing to the patient's developmental challenges. The study adhered to the ethical standards laid out in the Helsinki Declaration and received ethical approval from the University's Bioethics Committee under code 2024‐738. Written informed consent was obtained from the patient's guardians prior to participation. The consent included permission for the patient's participation in the research, the publication of the case report, and the use of any accompanying images or clinical data in accordance with ethical guidelines. All procedures performed in this study were in accordance with the ethical standards of the institutional and national research committee and with the 1964 Helsinki Declaration and its later amendments or comparable ethical standards.

## 3 Methods

### 3.1 Differential Diagnosis

The primary differential diagnosis was Primitive Reflex Integration Dysfunction, which was suggested by the persistence of multiple primitive reflexes beyond the typical age of integration. Other potential contributing factors, including neurodevelopmental disorders such as Autism Spectrum Disorder or Attention Deficit Hyperactivity Disorder, were ruled out through clinical assessments and patient history.

### 3.2 Examination

A thorough neurological clinical examination was conducted, focusing on the evaluation of primitive reflexes. The primitive reflexes evaluated included the Asymmetrical Neck Reflex, Symmetrical Neck Reflex, Tonic Labyrinthine Reflex (anterior and posterior), Spinal Galant Reflex, Grasp Reflex, Moro Reflex, and Paralysis by Fear Reflex. Each reflex was scored on a scale from 0 (integrated) to 4 (not integrated) [9].

#### 3.2.1 Asymmetrical Neck Reflex

This reflex is assessed by turning the head to one side, which causes the arm and leg on the same side to extend, while the opposite limbs flex. The patient was observed for limb movement and muscle tone changes in response to head turning.

#### 3.2.2 Symmetrical Neck Reflex

This reflex involves the flexion or extension of the neck, leading to corresponding movements in the arms and legs (e.g., neck flexion causes arm flexion and leg extension). The patient's limb movements were observed in response to neck flexion and extension.

#### 3.2.3 Tonic Labyrinthine Reflex—Anterior and Posterior

The anterior TLR is observed by tilting the head forward, which causes flexion of the limbs, while the posterior TLR is observed by tilting the head backward, causing limb extension. The patient was positioned to observe the limb responses to head tilting in both forward and backward directions.

#### 3.2.4 Spinal Galant Reflex

This reflex is tested by stroking the skin along the side of the spine, which should cause lateral flexion of the trunk toward the stimulated side. The patient's trunk movement was observed in response to stimulation along the spine.

#### 3.2.5 Grasp Reflex

This reflex is tested by placing an object in the palm of the hand, which should cause the fingers to close around it. The patient's grasping response was observed when an object was placed in their hand.

#### 3.2.6 Moro Reflex

This reflex is elicited by a sudden loss of support, causing the arms to spread out, then pull back in, and is usually followed by crying. The patient's reaction to a sudden change in head position was observed.

#### 3.2.7 Paralysis by Fear Reflex

This reflex is characterized by a freezing response when the patient perceives a sudden threat. The patient's reaction to sudden, loud noises was observed to determine the presence of the reflex.

All evaluations were carried out in a controlled environment, with a consistent temperature of 22.51°C ± 0.2°C and humidity of 42.1% ± 1.1%.

### 3.3 Treatment

In this study, participants received a customized functional neurology treatment based on the methodologies of NeuroReEvolution (http://nre‐therapy.com/). The treatment began with a detailed clinical assessment, including verbal and visual evaluations and specific neurology examinations of joints, to identify each participant's unique neurological issues. A certified professional with Level III certification in the Functional Neurology Manual Muscle Test from NeuroReEvolution provided personalized therapy tailored to correct these imbalances [7]. The treatment focused on enhancing proprioceptive reflexes, reducing trigger point sensitivity, and adopting a holistic approach. Proprioceptive reflexes, which respond to physical changes such as muscle stretching or tendon compression, are essential for posture, balance, and movement regulation. The therapy aimed to correct abnormalities in these reflexes that could cause discomfort or restrict movement. It also targeted dysfunctional mechanoreceptors and nociceptors, integrating the functional response by a blink reflex. This holistic approach considered the body as an integrated system, addressing both localized dysfunctions and related conditions that might impact overall neuromuscular health [5].

## 4 Conclusion and Results (Outcome and Follow‐Up)

The functional neurology intervention demonstrated significant improvements in the integration of primitive reflexes in the patient. Post‐treatment assessments showed that the Asymmetrical Neck Reflex improved from a score of 3 to 1, indicating partial integration. The Symmetrical Neck Reflex and both the anterior and posterior components of the Tonic Labyrinthine Reflex achieved full integration, with scores improving from 3 to 0 and 2 to 0, respectively. The Spinal Galant Reflex, which initially scored 1, was fully integrated (0), while the Grasp Reflex improved from 2 to 0, showing full integration. The Moro Reflex, which was already fully integrated before treatment, remained at 0, and the Paralysis by Fear Reflex improved significantly from 2 to 0.

These results highlight the effectiveness of a single session of functional neurology intervention in reducing the persistence of primitive reflexes, leading to improvements in motor coordination, balance, and cognitive function. The integration of these reflexes is critical for normal neurodevelopment, and the observed improvements suggest that functional neurology may be a viable treatment option for children with Primitive Reflex Integration Dysfunction.

### 4.1 Follow‐Up

While the results of this single session were promising, long‐term follow‐up assessments are recommended to determine the lasting effects of the intervention and to evaluate whether additional sessions may be necessary for complete reflex integration. Regular monitoring will ensure that the patient continues to benefit from the improvements observed and that any residual dysfunctions can be addressed promptly.

## 5 Discussion

Table 1 shows the improvement in the integration of primitive reflexes after the functional neurology intervention.

**TABLE 1:** Effectiveness of functional neurology treatment on primitive reflex integration.

Primitive reflex	Before treatment	After treatment
Asymmetrical neck reflex	3	1
Symmetrical neck reflex	3	0
Tonic labyrinthine reflex (anterior)	2	0
Tonic labyrinthine reflex (posterior)	2	0
Spinal galant reflex	1	0
Grasp reflex	2	0
Moro reflex	0	0
Paralysis by fear reflex	2	0

The functional neurology intervention led to significant improvements in the integration of the Asymmetrical Neck Reflex and the Symmetrical Neck Reflex. The ANR score decreased from 3 to 1, indicating a marked reduction in reflex activity. Similarly, the SNR score improved from 3 to 0, showing complete integration of the reflex. These changes suggest that the intervention effectively addressed the dysfunctions associated with these primitive reflexes. Previous studies have highlighted the importance of integrating primitive reflexes for optimal motor and cognitive development. For instance, the persistence of the ANR and SNR has been linked to issues in motor coordination and balance [10]. The improvement observed in this case aligns with findings from similar interventions, where functional neurology techniques were shown to facilitate reflex integration and enhance motor function [7]. The results of this study are consistent with the literature indicating that targeted neurological interventions can significantly impact reflex integration. In particular, the reduction in ANR activity parallels the findings of Harjpal and Kovela [4], who reported similar improvements in reflex integration following neuromodulatory treatments. However, the complete integration of the SNR in our study contrasts with some studies that observed only partial improvements, suggesting that the specific methodologies employed may play a crucial role in the outcomes [11]. These differences may be attributed to the personalized nature of the intervention used in this study, which included comprehensive assessments and tailored treatment plans.

The TLR encompasses both anterior and posterior components, each demonstrating significant changes following the functional neurology intervention. The anterior TLR, which involves flexion of the limbs when the head is tilted forward, improved from a score of 2 to 0. Similarly, the posterior TLR, characterized by limb extension when the head is tilted backward, also improved from 2 to 0. These results indicate complete integration of both reflexes post‐intervention. The TLR plays a crucial role in early motor development, influencing posture, balance, and coordination. Its persistence beyond infancy can lead to various developmental issues, including difficulties in motor control and spatial awareness [12]. The observed improvements align with previous findings that suggest targeted neurological interventions can facilitate the integration of primitive reflexes, thereby enhancing motor function [13]. Studies have shown that proper positioning and therapeutic interventions can significantly affect TLR integration. For example, research by Harjpal and Kovela [4] demonstrated that specific positioning could lead to improvements in TLR in children with cerebral palsy, supporting the efficacy of targeted interventions in reflex integration [4]. Our study's results are consistent with these findings, highlighting the potential of functional neurology to address and integrate retained reflexes effectively.

The functional neurology intervention produced notable changes in the Spinal Galant Reflex, Grasp Reflex, Moro Reflex, and Paralysis by Fear Reflex. The Spinal Galant Reflex showed complete integration, with the score improving from 1 to 0. This result aligns with previous findings that link successful intervention strategies to improved neuromotor outcomes. For instance, it was demonstrated that functional neurology approaches could effectively integrate the Spinal Galant Reflex, reducing related motor issues in children with learning disabilities, in line with previous interventions in neurological dysfunctions related to the visuovestibular system as saccadic, semicircular canals, vestibule‐ocular reflex, or even psychological dysfunctions as depression [14, 15, 16, 17]. The complete integration observed in our case supports the efficacy of functional neurology in addressing such reflex dysfunctions [9]. The Grasp Reflex also exhibited significant improvement, moving from a score of 2 to 0, indicating full integration. This reflex is crucial for developing fine motor skills and hand‐eye coordination [2]. The Paralysis by Fear Reflex, which improved from 2 to 0, shows full integration. This reflex, often associated with freezing responses to perceived threats, can significantly impact a child's ability to respond to stressors. Harjpal and Kovela [4] found that neuromodulatory treatments could significantly reduce the sensitivity of this reflex, thereby improving the child's adaptive responses to stress. Our findings corroborate this, highlighting the potential of functional neurology in mitigating the adverse effects of retained primitive reflexes.

### 5.1 Study Limitations and Future Research Lines

This study is limited by its single case report design, lack of a control group, and the potential for subjective bias in assessments, which restricts the generalizability of the findings. Additionally, the short duration precludes understanding the long‐term effects of the intervention. Future research should include larger sample sizes, randomized controlled trials, and longitudinal studies to validate the findings and assess long‐term outcomes. Exploring the mechanisms behind the interventions, comparing different neurological treatments, and incorporating objective measures like neuroimaging would provide a more comprehensive understanding of their impact.

### 5.2 Practical Applications

The findings of this study suggest several practical applications for functional neurology interventions in clinical settings. By effectively integrating primitive reflexes, such as the Asymmetrical Neck Reflex and the Symmetrical Neck Reflex, clinicians can address developmental issues related to motor coordination, balance, and cognitive function. The use of personalized treatment plans, including proprioceptive training and trigger point therapy, can enhance patient outcomes by targeting specific neurological dysfunctions. These interventions can be particularly beneficial for children with neurodevelopmental disorders, offering a holistic approach to improving their overall neuromuscular health and daily functioning.

## Author Contributions

**Jorge Rey‐Mota:** funding acquisition, methodology, writing – review and editing. **Guillermo Escribano‐Colmena:** conceptualization, supervision, writing – original draft. **Athanasios A. Dalamitros:** formal analysis, writing – original draft. **David Martín‐Caro Álvarez:** formal analysis, software, writing – original draft. **Vicente Javier Clemente‐Suárez:** formal analysis, funding acquisition, project administration, software, supervision, validation, visualization, writing – original draft, writing – review and editing.

## Ethics Statement

The study was conducted in accordance with the Declaration of Helsinki and approved by the university's ethics Committee of Universidad Europea de Madrid, assigning it an internal code 2024‐738.

## Consent

Written **i**nformed consent was obtained from the patient's guardians prior to participation.

## Conflicts of Interest

The authors declare no conflicts of interest. Guillermo Escribano‐Colmena and Jorge Rey‐Mota are affiliated with NeuroReEvolution.

## Data Availability

All data are presented in the manuscript.
